# Characteristics and clinical outcomes of culture-negative and culture-positive septic shock: a single-center retrospective cohort study

**DOI:** 10.1186/s13054-020-03421-4

**Published:** 2021-01-06

**Authors:** June-sung Kim, Youn-Jung Kim, Won Young Kim

**Affiliations:** Department of Emergency Medicine, University of Ulsan College of Medicine, Asan Medical Center, 88, Olympic-ro 43-gil., Songpa-gu, Seoul, 05505 Korea

**Keywords:** Sepsis, Septic shock, Mortality, Culture, Time to blood culture positivity

## Abstract

**Background:**

We evaluated the characteristics and outcomes of culture-negative versus culture-positive septic shock.

**Methods:**

We performed a retrospective observational study of data from a prospective registry from 2014 to 2018. A total of 2,499 adult patients with septic shock were enrolled. The primary outcome was 90-day mortality, and the secondary outcomes were the length of hospital stay, a requirement for mechanical ventilation or renal replacement therapy, and in-hospital mortality.

**Results:**

Of 1,718 patients with septic shock, 1,012 (58.9%) patients were culture-positive (blood 803, urine 302, sputum 102, others 204) and the median pathogen detection time was 9.5 h (aerobic 10.2 h and anaerobic 9.0 h). The most common site of culture-positive infection was the hepatobiliary tract (39.5%), while for the culture-negative it was the lower respiratory tract (38.2%). The culture-negative group had a lower mean body temperature (37.3 vs 37.7 ℃), lactate (2.5 vs. 3.2 mmol/L), C-reactive protein (11.1 vs 11.9 mg/dL), and sequential organ failure assessment score (7.0 vs. 8.0) than that of the culture-positive group. However, 90-day mortality between the groups was not significantly different (32.7 vs 32.2%, p = 0.83), and the other clinical outcomes also did not differ significantly. Moreover, a shorter culture detection time was correlated with a higher sequential organ failure assessment score but not with mortality.

**Conclusion:**

Patients with septic shock are frequently culture-negative, especially in cases where the infection focus is in the lower respiratory tract. Although culture-negative was associated with a degree of organ dysfunction, it was not an independent predictor of death.

## Introduction

The incidence of sepsis and septic shock has been increasing worldwide over the past decade, and its morbidity and mortality are still unacceptably high [1]. Early resuscitation for maintaining adequate tissue perfusion and the choice of proper antibiotics are mainstays to improve outcomes [2]. Although current guidelines recommend obtaining cultures before prescribing empirical antibiotics, in many cases, isolation of specific organisms by culture remains challenging. Previous studies have reported the proportion of culture-negative cases was between 28 and 49% of all patients with sepsis [3–5]; however, clinical outcomes between culture-negative and -positive patients have only been documented inconsistently and are controversial. Moreover, there is a remarkable paucity of investigations evaluating the characteristics and clinical outcomes of culture-negative patients with septic shock, especially among those visiting emergency departments.

Time-to-positivity (TPP) is defined as the duration of time from the start of incubation to the detection of bacterial growth by an automated culture system. It has been suggested as a prognostic marker of a fatal outcome. Numerous reports have revealed that a shorter TTP is associated with mortality among patients with each specific type of bacteremia [6, 7]. Septic shock is a heterogeneous syndrome, involving a variety of microorganisms and affecting numerous vital organs. However, the relationship between TTP and the entire sepsis entity is rarely reported.

We hypothesized that culture negativity was associated with worse outcomes than culture positivity because identifying the pathogens might help physicians choose the most appropriate antibiotics and determine the optimal duration of their use. Moreover, we presumed that an earlier detection time of culture could reflect the extent of bacterial loading and would be correlated with the severity of the illness. To prove this, we compared the clinical characteristics and outcomes of culture-negative septic shock (CNSS) cases versus culture-positive septic shock (CPSS) cases. We also evaluated the relationship between TTP and 90-day mortality in patients with septic shock.

## Methods

### Study design

We conducted a retrospective cohort study by analyzing data from a prospective septic shock registry at an urban tertiary emergency department with annual admissions of more than 120,000 patients from January 1, 2014, to December 31, 2018. This registry was designed to enhance internal quality improvement of septic shock patients visiting emergency departments and to manage multicenter cohort data of the Korean Shock Society for future analysis [8]. The Institutional Review Board of the study facility approved the study (no. 2016–0548) and waived the requirement for informed consent because of its retrospective characteristics.

### Data collection and definition of variables

This registry included all adult septic shock patients (≥ 18 years) consecutively diagnosed in the emergency department [9]. Infection was defined clinically by the emergency physicians on duty. In brief, they evaluated the systemic inflammatory response syndrome and applied the quick sequential organ failure assessment (SOFA) criteria for every patient with suspected or confirmed infection [9]. In addition, we used a definition of septic shock as refractory hypotension (mean arterial pressure ≤ 65 mmHg) requiring vasopressors despite adequate fluid infusion or a blood lactate level of at least 4 mmol/L, based on a previous definition [10]. This registry excluded those who were transferred from other hospitals after proper resuscitation, transferred to another hospital because of no room for admission, had a “do-not-resuscitate” order, or refused to accept treatment because of nonmedical issues, such as healthcare cost, fear of invasive procedures, or just not wanting to intensive care unit care (ICU) without definite reasons. We also excluded patients from this secondary analysis with microbiologically proven viral, fungal, and parasitic infections.

All enrolled patients were treated with protocol-driven resuscitation following the Surviving Sepsis Campaign guidelines [3]. In brief, aggressive fluids infusion and vasopressors were applied with blood pressure monitoring. Lactate levels were checked via venous or arterial blood gas analysis, and a central venous catheter was placed routinely for administering high-dose vasopressors and repetitive sampling [11, 12]. Blood cultures were obtained within 3 h of recognition from samples at two or more different anatomical sites according to the local practice [13]. When indwelling catheters were present, one blood sample was obtained through the catheter, and the remainder were taken from different peripheral venous sites. Site-specific cultures, including urine, sputum, pleural, ascites, stool, and pus, were performed following the physicians’ decisions. Broad-spectrum empirical antibiotics were infused as soon as possible, and percutaneous or endoscopic drainages were aggressively conducted after image work-ups. The sites of infection were grouped as the lower respiratory tract, urinary tract, gastrointestinal, and hepatobiliary systems. Minor sites, such as isolated blood stream infections, skin and soft tissue, and the central nervous system were categorized as others. The infectious sites were determined and recorded in the registry by the primary physicians on duty through the patients’ histories, physical examinations, and results of laboratory and imaging. We deemed fever without a definite focus as unknown.

Data regarding demographics, underlying diseases, initial vital signs, sites of infection, and clinical outcomes, such as intensive care unit (ICU) admission, requirements for a mechanical ventilator or renal replacement therapy, and duration of ICU stay and mechanical ventilation were obtained from the registry. The date of the patient’s death was obtained from the National Health Insurance Service in South Korea, and in-hospital mortality and 90-day mortality were extracted. Subgroup analysis of in-hospital mortality according to the site of infection was also investigated to confirm the clinical differences based on the infection sites. Laboratory examinations, including white blood cell counts, hemoglobin, prothrombin time (international normalization ratio), lactate, and C-reactive protein, were also extracted. Source control measures, including all physical procedures to eliminate the sources of the pathogen, including draining abscesses, debriding infected soft tissues, and extracting contaminated devices or foreign bodies, were recorded [14]. Escalation of antibiotics occurred whenever the initial antibiotics were changed to cover more extensive pathogens because of refractory shock or the identification of antibiotic-resistance pathogens. SOFA scores were calculated from the initial clinical and laboratory data on admission.

Moreover, microbiological culture results and the detection time of the enrolled patients were extracted from the electronic medical records and reviewed by three investigators (J.S.K., Y.J.K., and W.Y.K.). An experienced infection specialist made a final decision when there was a disagreement about discriminating the exact pathogen. Detection time was defined as the time to positive detection of the pathogens and was calculated by subtracting the time of receipt in the laboratory from the time required to detect a positive culture. When growth was detected in both aerobic and anaerobic bottles, each time was recorded [see Additional file 1].

The primary outcome was 90-day mortality, and the secondary outcomes were ICU admission, ICU length of stay, mechanical ventilator or renal replacement therapy requirement, mechanical ventilation duration, and in-hospital mortality.

## Statistical analysis

Descriptive statistics were stratified by culture results (i.e., culture-positive and -negative). Baseline demographics, clinical characteristics, and outcomes are presented as the frequency and percentage for categorical and median with interquartile range (IQR) for continuous variables. The Kolmogorov–Smirnov test was used to check the normality of the distribution. Categorical variables were analyzed using Chi-squared or Fisher’s exact tests. Univariate logistic regression tests were performed with potential risk factors, which showed differences between two groups. Multivariate logistic regression was conducted with the variables that had significance in the univariate logistic regression analysis. The absence of multicollinearity was confirmed by using regression analysis with calculating the variance inflation factor values. Kaplan–Meier survival curves with the log-rank test were stratified by the culture results. The correlations between the severity of the septic shock and culture detection time were assessed by the Spearman rank correlation coefficient. We considered *P* values less than 0.05 as statistically significant. Analyses were performed using SPSS Statistics for Windows, version 26 (IBM Corp., Armonk, NY, USA).

## Results

**Fig. 1:**
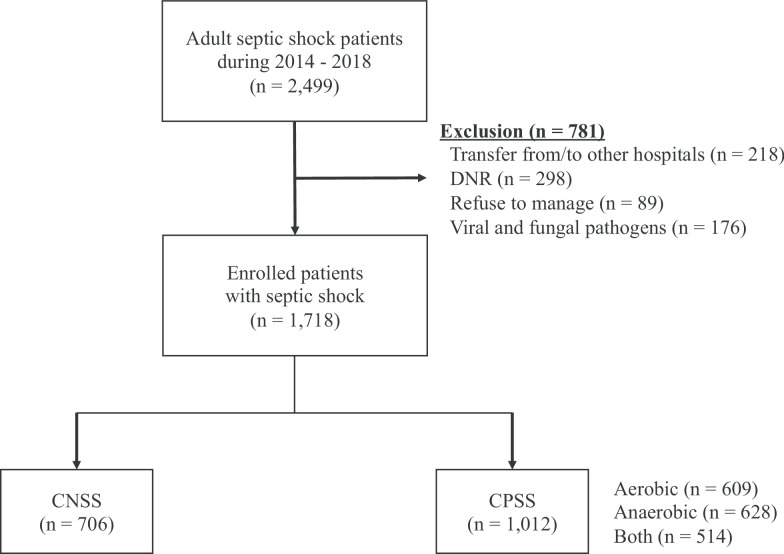
Flowchart of the study population. Prospective septic shock registry excluded 605 patients (218 for transfer from/to other hospitals, 298 for do-not-resuscitate orders, 89 for refuse to manage), and 176 patients with proven viral and fungal pathogen were also precluded from this analysis

During the study period, 2,499 adult septic shock patients visited the emergency department, and 781 were not included in this study due to the exclusion criteria (218 transferred from/to other hospitals, 298 had do-not-resuscitate orders, 89 refused treatment, and 176 had known viral or fungal pathogens) (Fig. 1). Of the 1,718 cases, 706 (41.1%) were in the CNSS and 1,012 (58.9%) were in the CPSS group.

Table 1 presents the baseline characteristics of the study population according to the culture results. The CPSS group tended to be older and had a higher proportion of female patients. All underlying diseases, except chronic kidney diseases (10.9 vs. 18.1%, *P* < 0.01; CPSS vs. CNSS, respectively), were similar between the groups. Patients who were pathogen-positive showed slightly lesser tachypnea and a higher mean body temperature than those who were pathogen-negative. In the laboratory results, initial lactate (3.2 vs. 2.5 mmol/L, *P* < 0.01) and total bilirubin (1.3 vs. 0.9 mg/dL, *P* < 0.01) were elevated more in the CPSS than in the CNSS patients. Furthermore, the initial SOFA score (8.0 vs. 7.0, *P* < 0.01) was higher, and source control (44.6 vs. 28.6%, *P* < 0.01) and the escalation of antibiotics (15.6 vs. 4.4%, *P* < 0.01) were more frequent in patients with CPSS.

**Table 1:** Baseline characteristics of septic shock according to the culture results

Characteristics	Total (N = 1,718)	CNSS (N = 706)	CPSS (N = 1,012)	*P*
Age	66.0 (58.0–74.0)	66.0 (56.0–74.0)	68.0 (59.0–74.0)	0.02
Male	1,035 (60.2)	450 (63.7)	585 (57.8)	0.01
Past illness				
HTN	605 (35.2)	261 (37.0)	344 (34.0)	0.20
DM	425 (24.7)	165 (23.4)	260 (25.7)	0.27
CAD	182 (10.6)	72 (10.2)	110 (10.9)	0.66
Pulmonary disease	108 (6.3)	49 (6.9)	59 (5.8)	0.36
Malignancy	782 (45.5)	302 (42.8)	480 (47.4)	0.06
Hematologic disorder	118 (6.9)	53 (7.5)	65 (6.4)	0.39
CKD	238 (13.9)	128 (18.1)	110 (10.9)	< 0.01
LC	29 (1.7)	9 (1.3)	20 (2.0)	0.34
Initial vital signs				
SBP (mmHg)	91.0 (80.0–112.0)	90.0 (80.0–109.0)	93.0 (80.0–113.0)	0.28
DBP (mmHg)	58.0 (50.0–69.0)	58.0 (50.0–68.0)	58.0 (50.0–71.0)	0.73
PR (/min)	107.0 (89.0–125.0)	105.0 (88.0–123.0)	108.0 (90.0–126.0)	0.06
RR (/min)	20.0 (20.0–24.0)	20.0 (20.0–24.0)	20.0 (20.0–22.0)	0.04
BT (℃)	37.6 (36.6–38.7)	37.2 (36.5–38.2)	37.7 (36.7–38.9)	< 0.01
Laboratory				
WBC (× 10^3^/μL)	9.7 (5.0–15.9)	10.2 (5.5–16.2)	9.2 (4.7–15.7)	0.06
Hemoglobin (g/dL)	10.7 (9.1–12.4)	10.7 (9.2–12.6)	10.7 (9.0–12.3)	0.30
PT (INR)	1.3 (1.1–1.5)	1.3 (1.1–1.4)	1.3 (1.1–1.5)	0.20
Lactate (mmol/L)	2.9 (1.7–5.1)	2.5 (1.5–4.5)	3.2 (1.9–5.4)	< 0.01
BUN (mg/dL)	25.0 (17.0–38.0)	24.0 (17.0–39.0)	25.0 (17.0–37.0)	0.95
Creatinine (mg/dL)	1.3 (0.9–2.1)	1.3 (0.9–2.2)	1.3 (0.9–2.1)	0.10
Bilirubin (mg/dL)	1.1 (0.6–2.4)	0.9 (0.5–1.7)	1.3 (0.7–3.0)	< 0.01
Albumin (g/dL)	2.6 (2.2–3.1)	2.6 (2.2–3.1)	2.6 (2.2–3.1)	0.37
CRP (mg/dL)	11.6 (5.0–19.8)	11.1 (4.6–19.2)	11.9 (5.3–20.2)	0.02
SOFA score	7.0 (5.0–10.0)	7.0 (5.0–10.0)	8.0 (6.0–10.0)	< 0.01
Source control	653 (38.0)	202 (28.6)	451 (44.6)	< 0.01
Antibiotics escalation	189 (11.0)	31 (4.4)	158 (15.6)	< 0.01

Data are presented as n (%) or mean with standard deviation

Abbreviations: CNSS, culture negative septic shock; CPSS, culture positive septic shock; HTN, hypertension; DM, diabetes mellitus; CAD, coronary artery disease; CKD, chronic kidney disease; LC, liver cirrhosis; SBP, systolic blood pressure; DBP, diastolic blood pressure; PR, pulse rate; RR, respiratory rate; BT, body temperature; GCS, Glasgow Coma Scale; WBC, white blood cells; PT, prothrombin time; INR, international normalized ratio; BUN, blood urea nitrogen; CRP, C-reactive protein; and SOFA, sequential organ failure assessment

*Escherichia coli* was the most common causative organism, followed by *Klebsiella* species (including *Klebsiella pneumoniae*, *oxytoca*, and *ornithinolytica*) (Table 2). Gram-positive bacteria, such as *Staphylococcus* and *Streptococcus*, were less frequent than Gram-negative bacteria. An additional table shows this in more detail [see Additional file 2].

**Table 2:** Frequency of bacteria for the culture-positive septic shock

Species	Frequency (%)
*Escherichia coli*	377 (37.3)
*Klebsiella* species	200 (19.8)
*Staphylococcus* species	83 (8.2)
*Streptococcus* species	63 (6.2)
*Enterococcus* species	57 (5.6)
*Enterobacter* species	53 (5.2)
*Pseudomonas aeruginosa*	51 (5.0)
*Citrobacter freundii*	21 (2.1)
*Acinetobacter baumannii*	20 (2.0)
*Clostridium* species	14 (1.4)
Etc.^a^	71 (7.0)

Data are presented as n (%)

^a^Etc included *Bacillus* species, *Bacteroides* species, *Campylobacter* species, *Chryseobacterium* indologenes, *Eggerthella lenta*, *Flayonifractor plautii*, *Fusobacterium* species, *Haemophilus influenzae*, *Kocuria* species, *Morganella morganii*, *Peptostreptococcus* species, *Proteus mirabilis*, *Vibrio vulnificus*

**Fig. 2:**
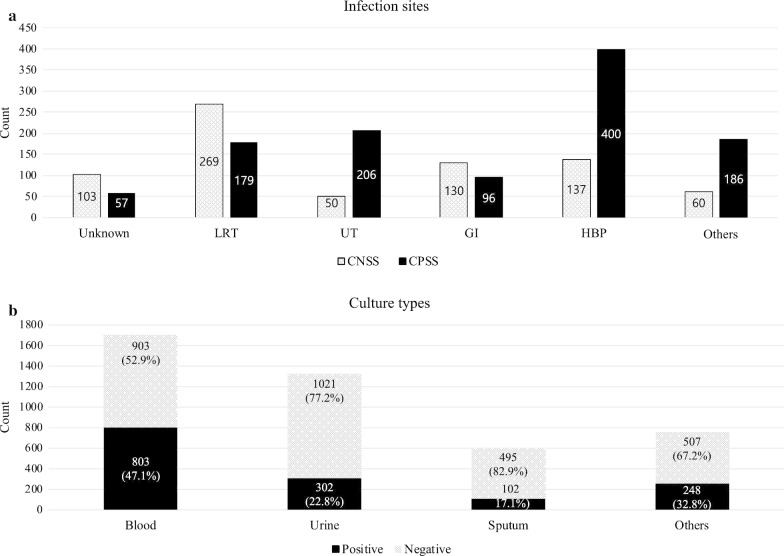
Frequencies of infection foci (**a**) and the culture types (**b**) by the culture results. **a** Isolated blood stream infection, skin and soft tissue, and central nervous system were categorized as “other”. **b** Other culture types included pleural, ascites, stool, and pus. Abbreviations: LRT = lower respiratory tract; UT = urinary tract; GI = gastro-intestinal; HBP = hepatobiliary-pancreas

Figure 2 shows the frequencies of infection focuses and culture types according to the culture results. Lower respiratory tract and gastrointestinal focuses were the most frequent sites in the negative culture cases; meanwhile, the urinary tract and hepatobiliary pancreas were the most common sites among the positive culture cases. Blood cultures were predominantly performed and showed the highest positive rates (47.1%) followed by the others (32.8%), including pleural effusion, ascites, open pus, and closed pus obtained by endoscopic or percutaneous drainage.

**Fig. 3:**
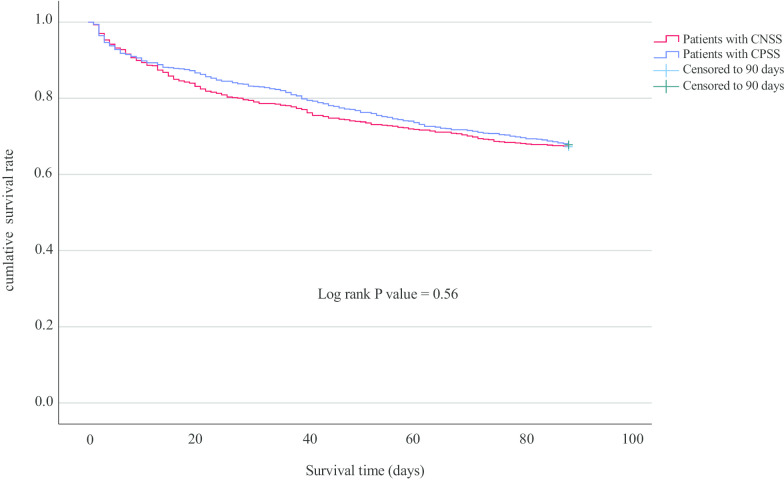
Comparison of Kaplan–Meier survival curves between patients with culture-negative (CNSS) and culture-positive septic shock (CPSS)

For the clinical outcomes, both groups had similar ICU admission rates, ICU length of stay, and requirements for renal replacement therapy (Table 3). The CNSS patients had higher requirements for mechanical ventilators; however, the median duration of mechanical ventilator use was not different between the groups. Moreover, multivariate logistic regression was performed for the variables that were statistically different in the univariate analysis [see Additional file 3], and the effect of culture positivity on the requirements for mechanical ventilators disappeared after adjusting for the confounders [see Additional file 4]. There was no multicollinearity among the variables, especially between the lactate level and SOFA score (variance inflation factors 1.084). The in-hospital and 90-day mortalities were similar in both groups. Subgroup analysis according to the sites of infection showed that patients with lower respiratory infections had the highest in-hospital mortality (38%) [see Additional file 5]. Figure 3 represents a comparison of 90-day survivor curves between patients with CNSS and CPSS. They showed similar 90-day survival rates (log-rank *P* = 0.56).

**Table 3:** Comparison of the clinical outcomes for both culture-negative and culture-positive septic shock

	Total (n = 1,718)	CNSS (n = 706)	CPSS (n = 1,012)	*P*
ICU admission	818 (47.6)	321 (45.5)	497 (49.1)	0.14
ICU stay (days)	4.0 (3.0–10.0)	5.0 (3.0–10.0)	4.0 (3.0–10.0)	0.13
RRT requirements	225 (13.1)	81 (11.5)	144 (14.2)	0.29
MV requirements	411 (23.9)	186 (26.3)	225 (22.2)	0.05
MV duration (days)	7.0 (3.0–13.0)	7.0 (3.0–12.0)	7.0 (3.0–14.0)	0.45
In-hospital mortality	293 (17.1)	123 (17.4)	170 (16.8)	0.75
90-day mortality	557 (32.4)	231 (32.7)	326 (32.2)	0.83

Data are presented as median with interquartile ranges

Abbreviations: CNSS, culture-negative septic shock; CPSS, culture-positive septic shock; ICU, intensive care unit; RRT, renal replacement therapy; and MV, mechanical ventilation

**Fig. 4:**
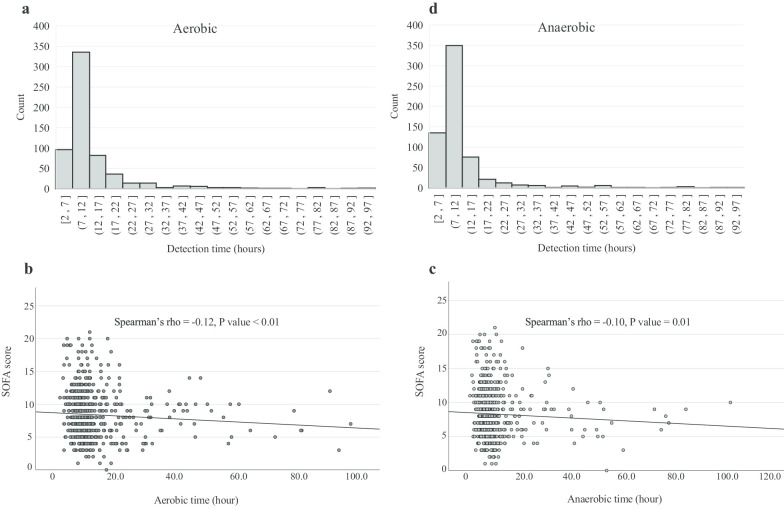
Distributions and scatter plots of the first detected culture-positive time of the aerobic and anaerobic pathogens. Time-to-positivity in both aerobic (**a**) and anaerobic (**b**) pathogens. Scatter plots of aerobic (**c**) and anaerobic (**d**) time according to sequential organ failure assessment (SOFA)

The median TPP for the culture-positive pathogens detection time was 9.5 h (aerobic 10.2 h and anaerobic 9.0 h), and most of the aerobic and anaerobic detection times were within 16 h (Fig. 4). Although the 90-day survivor and nonsurvivor groups had similar detection times (9.6 [IQR 8.1–11.9] vs. 9.4 h [IQR 8.0–12.2] for aerobe; 8.4 [IQR 6.8–10.1] vs. 8.4 h [IQR 6.8–11.0] for anaerobe), the shorter TPP of both aerobe and anaerobe was correlated with higher SOFA scores (Spearman’s rho = − 0.12, p < 0.01 for aerobe; Spearman’s rho = − 0.10, p = 0.01 for anaerobe) (Fig. 4).

## Discussion

Our results demonstrated that (1) about 41% of patients with septic shock were culture-negative; (2) patients with CNSS showed similar in-hospital and 90-day mortality as those with CPSS; and (3) a shorter detection time of both aerobes and anaerobes was correlated with a higher SOFA score but not with mortality.

This study found that negative culture results were quite common in septic shock patients, which is supported by the findings of a recent retrospective study using nationwide data covering ten years that revealed that the incidence of culture-negative status among patients with severe sepsis was increasing annually by about 28% [15]. The reasons for the continuous increment of culture negativity could be multifactorial. First, the patients could have been prescribed empirical antibiotics at local clinics before sepsis developed [16]. Second, the proportion of sepsis cases caused by atypical pathogens, including viral and fungal infections, might be increasing [17, 18]. In these cases, conventional cultures, such as blood, urine, stool, and pus swabs, were not enough to detect the pathogens, but definitive evaluations could be helpful. For example, our results showed that sputum cultures had a quite low positivity rate, and bronchial aspiration could enhance the possibility of identifying the causative pathogens [19]. Third, some of the patients with negative cultures had sepsis that was the result of noninfectious causes, such as metabolic disorders, inflammatory diseases, adverse effects of drugs, or malignancies. However, the proportion of those mimics would not be significant and not change the results because all of the data in this registry were reviewed retrospectively by experienced physicians before the final analysis.

Our study also found that CNSS behaved quite similarly to CPSS. Looking at the literature, Gupta et al. reported that severe sepsis cases with negative cultures had more comorbidities and organ failure, and it was an independent predictor of death after adjusting for confounders (OR 1.75 [95% CI 1.72–1.77]) [15]. On the other hand, two retrospective studies found that culture-negative and culture-positive severe sepsis had similar outcomes after adjusting for confounders, such as demographics, the site of infection, and appropriate antibiotics [20, 21]. These differences are likely due to differences in patient populations, proportions of the sites of infection, and resistance of the bacteria to antibiotics. For example, our study included much older patients (mean 66.0 vs. 63.0 years) with higher proportion of malignancies (45.5 vs. 7.0%) compared with the retrospective study of Phua et al. [20]. Furthermore, the sites of infection were also quite different between the studies. Because sepsis is a heterogeneous syndrome, infectious sites with specific organisms may have totally different clinical characteristics. One multicenter, prospective study in Japan supported this assumption that the clinical characteristics and outcomes of patients with severe sepsis and septic shock varied according to the sites of infection [22]. Their finding was also consistent with ours that urinary tract infections were associated with less in-hospital mortality than that of the others [see Additional file 5]. Interestingly, lower respiratory and intra-abdominal infections were predominant in our culture-negative group, which suggests the site of infection may be associated with CNSS. Previous retrospective studies found that culture-positive patients with lung and intra-abdominal infections were associated with poor outcomes [23, 24]. The authors thought that culture negativity might imply susceptibility to the initial antibiotic regimens prescribed, leading to a lesser severity of illness. On the other hand, subgroup analyses of our study showed higher mortality among CNSS with lung infections that could lead to different outcomes, suggesting that the clinical outcomes may be associated with not only the infection sources but also the management of the shock.

We also explored the correlation between time to blood culture positivity and outcomes. TPP may provide practical information because a short incubation time can reflect a higher bacterial load with greater virulence [25]. Moreover, TPP has a prognostic role in some specific disease entities, such as catheter-related bloodstream infections and infective endocarditis [26, 27]. One notable finding was that the TTPs of both the aerobic and anaerobic bottles were distributed within 16 h in the CPSS, which was significantly shorter than that of a prior study of *Staphylococcus aureus* bacteremia (11.0 vs. 21.8 h) [28]. Septic shock, the most severe form of infection, generally presents with a higher bacterial load. In contrast to our initial hypothesis, we found that a quick detection time of the microorganism was correlated with greater severity of the disease with a higher SOFA score, but not with mortality. These results could be interpreted to mean that although a faster TTP could imply a high bacterial load, a shorter time could give more timely information about the antibiotics susceptibility and help with the selection of the proper antimicrobial agents [29].

## Limitations

We noticed several limitations to our study. First, the study results were derived from a single institution retrospectively, which therefore limited its generalizability to other populations. Second, there could be unmeasured confounders, including the selection, timing, and duration of antibiotics, which might lead to different outcomes. However, we tried to include numerous well-known variables related to the outcomes of sepsis in the multivariate analysis, such as age, lactate, source control, and SOFA score. Third, some culture-negative results were because of inappropriate sampling. Nonetheless, most culture samples were obtained by experienced nurses and physicians, and this was less likely to be a significant confounder.

## Conclusion

In conclusion, our study confirmed that about 40% of patients with septic shock are culture-negative. CNSS patients had similar clinical outcomes as those who had CPSS, which means CNSS and CPSS status did not demonstrate prognostic value in our investigation. Moreover, an early detection time to culture positivity is not associated with mortality.

## Supplementary information

**Additional file 1**. ** Figure 1**: Septic shock registry of the study population. Emergency medicine (EM) physicians on duty recognized the patients with presumed septic shock consecutively. They resuscitated patients and enrolled them in the registry with informed consent. All of the data were reviewed by the well-trained EM staff who majored in critical care and decided on the final inclusion. After excluding septic shock-mimic cases, EM physicians collected numerous variables, including demographic data, laboratory data, radiologic results, treatment-related parameters, and clinical outcomes. For the secondary analysis, investigators who were involved this study extracted additional data, such as culture results, and time-to-positivity.

**Additional file 2**. ** Table 1**: Frequency of the subspecies of the isolated bacteria.

**Additional file 3**. ** Table 2**: Baseline characteristics of septic shock according to the culture results.

**Additional file 4**. ** Table 3**: Univariate and multivariate analysis for predicting mechanical ventilator requirements.

**Additional file 5**. **Table 4**: In-hospital mortality according to sites of infection among patients with culture-negative septic shock.

## Data Availability

The datasets generated and analyzed during the current study are available from the corresponding author on reasonable request.

## Abbreviations

CNSS: Culture-negative septic shock
CPSS: Culture-positive septic shock
DNR: Do-not-resuscitate
ICU: Intensive care unit
IQR: Interquartile range
SOFA: Sequential organ failure assessment
TTP: Time-to-positivity
