# Supplementary figures and images for: Characteristics and clinical outcomes of culture-negative and culture-positive septic shock: a single-center retrospective cohort study
Source: Crit Care. 2021 Jan 6;25:11. doi: 10.1186/s13054-020-03421-4 (PMC7787242; doi:10.1186/s13054-020-03421-4)

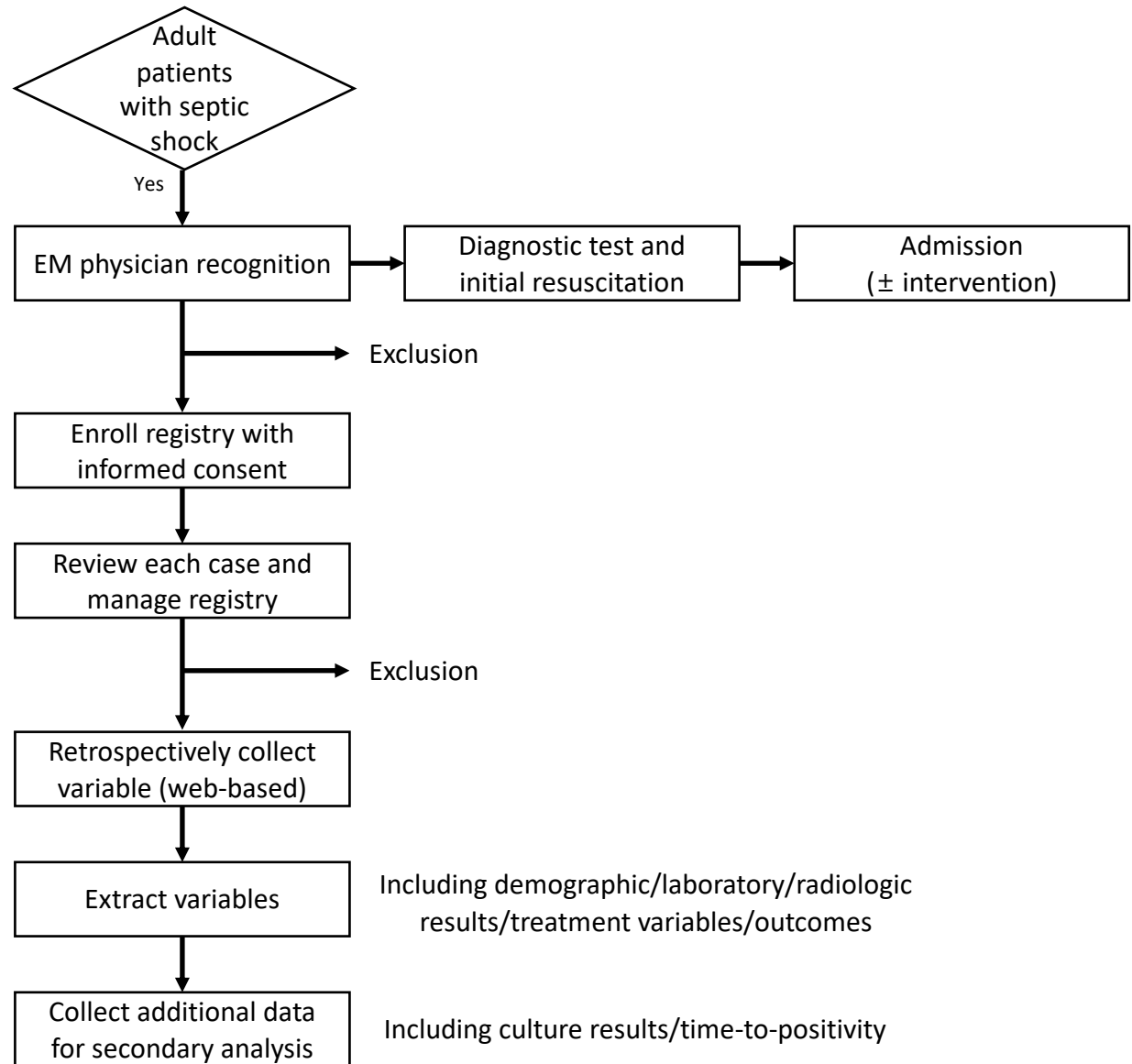

Supplement: Supplementary file 1 — Additional file 1. Figure 1: Septic shock registry of the study population. Emergency medicine (EM) physicians on duty recognized the patients with presumed septic shock consecutively. They resuscitated patients and enrolled them in the registry with informed consent. All of the data were reviewed by the well-trained EM staff who majored in critical care and decided on the final inclusion. After excluding septic shock-mimic cases, EM physicians collected numerous variables, including demographic data, laboratory data, radiologic results, treatment-related parameters, and clinical outcomes. For the secondary analysis, investigators who were involved this study extracted additional data, such as culture results, and time-to-positivity. [file 13054_2020_3421_MOESM1_ESM.pdf]
