# Supplementary material for: Characteristics and clinical outcomes of culture-negative and culture-positive septic shock: a single-center retrospective cohort study
Source: Crit Care. 2021 Jan 6;25:11. doi: 10.1186/s13054-020-03421-4 (PMC7787242; doi:10.1186/s13054-020-03421-4)
Supplement: Supplementary file 2 — Additional file 2. Table 1: Frequency of the subspecies of the isolated bacteria. [file 13054_2020_3421_MOESM2_ESM.pdf]

Supplementary Table 1. Frequency of the sub-species of the isolated bacteria

Species	Frequency (%)
Escherichia coli	377 (37.3)
Klebsiella species	200 (19.8)
Klebsiella pneumoniae	172
Klebsiella oxytoca	26
Klebsiella aerogenes	2
Staphylococcus species	83 (8.2)
Staphylococcus aureus	55
Staphylococcus epidermidis	17
Staphylococcus capitis	7
Other Coagulase-negative *Staphylococci*[1]	4
Streptococcus species	63 (6.2)
Streptococcus anginosus	17
Streptococcus pneumoniae	15
Streptococcus agalactiae	11
Other *Streptococci*[2]	20
Enterococcus species[3]	57 (5.6)
Enterobacter species[4]	53 (5.2)
Pseudomonas aeruginosa	51 (5.0)
Citrobacter freundii	21 (2.1)
Acinetobacter baumannii	20 (2.0)
Clostridium	14 (1.4)
Etc.[5]	71 (7.0)

Data are presented as n (%).

[1]Other Coagulase-negative *Staphylococci* included *Stap. haemolyticus*, *Stap. lugdunesis*, *Stap. saccharolyticus*.

[2]Other *Streptococci* included *Strep. constellatus*, *Strep. dysgalactiae*, *Strep. pyogenes*.

[3]*Enterococcus* species contained *E. faecium*, *E. faecalis*, *E. casseliflavus*, *E. gallinarum*, *E. raffinosus*.

[4]*Enterobacter* species contained *E. faecium*, *E. cloacae*, *E. aerogenes*, *E. avium*, *E. kobei*, *E. sakazakii*.

[5]Et cetera included *Bacillus* species, *Bacteroides* species, *Campylobacter* species, *Chryseobacterium* indologenes, *Eggerthella lenta*, *Flayonifractor plautii*, *Fusobacterium* species, *Haemophilus influenzae*, *Kocuria* species, *Morganella morganii*, *Peptostreptococcus* species, *Proteus mirabilis*, *Vibrio vulnificus*.
