# Supplementary material for: Characteristics and clinical outcomes of culture-negative and culture-positive septic shock: a single-center retrospective cohort study
Source: Crit Care. 2021 Jan 6;25:11. doi: 10.1186/s13054-020-03421-4 (PMC7787242; doi:10.1186/s13054-020-03421-4)
Supplement: Supplementary file 3 — Additional file 3. Table 2: Baseline characteristics of septic shock according to the culture results. [file 13054_2020_3421_MOESM3_ESM.pdf]

Supplementary Table 2. Baseline characteristics of septic shock according to the culture results

Characteristics	Total (N = 1,718)	Not required MV (N = 1,307)	Required MV (N = 411)	*P*
Age	66.0 (58.0 – 74.0)	66.0 (59.0 – 76.0)	67.0 (59.0 – 75.0)	< 0.01
Male	1,035 (60.2)	769 (58.8)	266 (64.7)	0.03
Past illness				
HTN	605 (35.2)	457 (35.0)	148 (36.0)	0.70
DM	425 (24.7)	318 (24.3)	107 (26.0)	0.49
CAD	182 (10.6)	120 (9.2)	62 (15.1)	< 0.01
Pulmonary disease	108 (6.3)	76 (5.8)	32 (7.8)	0.15
Malignancy	782 (45.5)	631 (48.3)	151 (36.7)	< 0.01
Hematologic disorder	118 (6.9)	79 (6.0)	39 (9.5)	0.02
CKD	238 (13.9)	150 (11.5)	88 (21.4)	< 0.01
LC	29 (1.7)	20 (1.5)	9 (2.2)	0.37
Sites of infection				
Unknown	160 (9.3)	133 (10.2)	27 (6.6)	0.03
LRT	448 (26.1)	232 (17.8)	216 (52.6)	< 0.01
Urinary tract	256 (14.9)	224 (17.1)	32 (7.8)	< 0.01
Intra-abdomen	226 (13.2)	172 (13.2)	54 (13.1)	0.99
Hepato-biliary	537 (31.3)	477 (36.5)	60 (14.6)	< 0.01
Others	61 (3.6)	44 (3.4)	17 (4.1)	0.46
Laboratory				
WBC ($\times 10^3/\mu L$)	9.7 (5.0 – 15.9)	11.4 (6.0 – 18.9)	10.1 (4.6 – 16.2)	0.57

Hemoglobin (g/dL)	10.7 (9.1 – 12.4)	11.0 (9.0 – 12.9)	11.3 (9.2 – 13.1)	< 0.01
PT (INR)	1.3 (1.1 – 1.5)	1.2 (1.1 – 1.4)	1.3 (1.2 – 1.5)	< 0.01
Lactate (mmol/L)	2.9 (1.7 – 5.1)	2.9 (1.9 – 4.6)	4.9 (2.8 – 7.5)	< 0.01
BUN (mg/dL)	25.0 (17.0 – 38.0)	27.0 (18.0 – 38.0)	32.0 (23.0 – 51.0)	< 0.01
Creatinine (mg/dL)	1.3 (0.9 – 2.1)	1.5 (1.0 – 2.4)	1.6 (1.1 – 3.0)	< 0.01
Albumin (g/dL)	2.6 (2.2 – 3.1)	2.6 (2.2 – 3.1)	2.5 (2.1 – 3.0)	< 0.01
CRP (mg/dL)	11.7 (5.0 – 19.8)	13.2 (6.7 – 20.9)	14.6 (6.2 – 23.5)	< 0.01
SOFA score	7.0 (5.0 – 10.0)	7.0 (5.0 – 10.0)	12.0 (9.0 – 15.0)	< 0.01
Source control	653 (38.1)	494 (37.8)	157 (38.2)	< 0.01
Antibiotics escalation	189 (12.5)	149 (13.0)	40 (11.0)	0.32
Culture positivity	1,012 (58.9)	787 (60.2)	225 (54.7)	0.04

Data are presented as n (%) or mean with standard deviation.

Abbreviations: MV, mechanical ventilator; HTN, hypertension; DM, diabetes mellitus; CAD, coronary artery disease; CKD, chronic kidney disease; LC, liver cirrhosis; WBC, white blood cells; PT, prothrombin time; INR, international normalized ratio; BUN, blood urea nitrogen; CRP, C-reactive protein; SOFA, sequential organ failure assessment.
