# Supplementary material for: Characteristics and clinical outcomes of culture-negative and culture-positive septic shock: a single-center retrospective cohort study
Source: Crit Care. 2021 Jan 6;25:11. doi: 10.1186/s13054-020-03421-4 (PMC7787242; doi:10.1186/s13054-020-03421-4)
Supplement: Supplementary file 4 — Additional file 4. Table 3: Univariate and multivariate analysis for predicting mechanical ventilator requirements. [file 13054_2020_3421_MOESM4_ESM.pdf]

Supplementary Table 3. Univariate and multivariate analysis for predicting mechanical ventilator requirement

	Univariate			Multivariate		
	OR	95% CI	*p*	Adjusted OR	95% CI	*p*
Male	1.28	1.02–1.61	0.03			
CAD	1.76	1.27–2.44	< 0.01			
CKD	2.10	1.57–2.81	< 0.01			
Malignancy	1.63	1.09–2.43	0.02			
Unknown	0.62	0.40–0.95	0.95			
LRT infection	5.13	4.04–6.53	< 0.01	5.14	2.86–9.23	< 0.01
UT infection	0.41	0.28–0.60	< 0.01	0.34	0.15–0.75	< 0.01
HBP infection	0.30	0.22–0.40	< 0.01	0.28	0.16–0.48	< 0.01
CPSS	0.80	0.64–0.99	0.05			
Age	1.02	1.00–1.03	0.02			
Hemoglobin	1.17	1.09–1.26	< 0.01			
BUN	1.01	1.00–1.02	< 0.01			
Creatinine	1.01	0.93–1.10	0.76			
Albumin	1.02	0.91–1.16	0.69	1.23	0.87–1.73	0.25
Lactate	1.20	1.13–1.26	< 0.01	1.17	1.09–1.26	< 0.01
SOFA score	1.41	1.33–1.49	< 0.01	1.31	1.22–1.42	< 0.01

Abbreviations: OR = odds ratio; CI = confidence interval; CAD = coronary artery disease; CKD = chronic kidney disease; LRT = lower respiratory tract; UT = urinary tract; HBP = hepatobiliary-pancreas; CPSS = culture-positive septic shock; BUN = blood urine nitrogen; SOFA = sequential organ failure assessment.
