# Supplementary material for: Characteristics and clinical outcomes of culture-negative and culture-positive septic shock: a single-center retrospective cohort study
Source: Crit Care. 2021 Jan 6;25:11. doi: 10.1186/s13054-020-03421-4 (PMC7787242; doi:10.1186/s13054-020-03421-4)
Supplement: Supplementary file 5 — Additional file 5. Table 4: In-hospital mortality according to sites of infection among patients with culture-negative septic shock. [file 13054_2020_3421_MOESM5_ESM.pdf]

Supplementary Table 4. In-hospital mortality according to sites of infection among patients with culture-negative septic shock

Site of infection	Total (N = 706)	Survival (N = 590)	In-hospital death (N = 116)	*P*
Unknown	103 (14.6)	87 (14.7)	16 (13.8)	0.79
Low respiratory tract	269 (38.1)	199 (33.7)	70 (60.3)	< 0.01
Urinary tract	50 (7.1)	46 (7.8)	4 (3.4)	0.10
Gastro-intestinal	130 (18.4)	112 (19.0)	18 (15.5)	0.38
Hepatobiliary-pancreas	137 (19.4)	125 (21.2)	12 (10.3)	< 0.01

Data are presented as n (%).
